# Regulation of Dendritic Cell Function by Vitamin D

**DOI:** 10.3390/nu7095383

**Published:** 2015-09-21

**Authors:** Myriam Barragan, Misty Good, Jay K. Kolls

**Affiliations:** 1 Richard King Mellon Foundation Institute for Pediatric Research, Children’s Hospital of Pittsburgh of UPMC, Pittsburgh, PA 15224, USA; E-Mails: Myriam.Barragan@bcm.edu (M.B.); goodml3@upmc.edu (M.G.); 2 Division of Gastroenterology, Hepatology and Nutrition, Children’s Hospital of Pittsburgh of UPMC, Pittsburgh, PA 15224, USA; 3 Department of Pediatrics, School of Medicine, University of Pittsburgh Pittsburgh, PA 15224, USA; 4 Division of Newborn Medicine, School of Medicine, University of Pittsburgh, Pittsburgh, PA 15224, USA

**Keywords:** vitamin D, vitamin D receptor, dendritic cells, innate and adaptive immunity, interleukins, cytokines, inflammation

## Abstract

Studies over the last two decades have revealed profound immunomodulatory aspects of vitamin D on various aspects of the immune system. This review will provide an overview of Vitamin D metabolism, a description of dendritic cell subsets, and highlight recent advances on the effects of vitamin D on dendritic cell function, maturation, cytokine production and antigen presentation. The active form of vitamin D, 1,25(OH)_2_D_3_, has important immunoregulatory and anti-inflammatory effects. Specifically, the 1,25(OH)_2_D_3_-Vitamin D_3_ complex can affect the maturation and migration of many dendritic cell subsets, conferring a special immunoregulatory role as well as tolerogenic properties affecting cytokine and chemokine production. Furthermore, there have been many recent studies demonstrating the effects of Vitamin D on allergic disease and autoimmunity. A clear understanding of the effects of the various forms of Vitamin D will provide new opportunities to improve human health.

## 1. Overview of Vitamin D Metabolism

Vitamin D plays a key role in maintaining mineral homeostasis. However, over the last several years, non-classic actions of vitamin D have been described. There are two main sources of vitamin D, including dietary intake and its synthesis in the skin exposed to sunlight [1]. During sunlight exposure, 7-dehydrocholesterol (7-DHC) in the skin is converted to the previtamin precholecalciferol that is then converted into activated 7-dehydrocholesterol or vitamin D_3_ [2,3]. Dietary or cutaneous vitamin D has to undergo two metabolic modifications in the liver and kidney to be converted into the bio-active form [4]. Vitamin D_3_ is transported to the liver where it undergoes hydroxylation by the enzyme 25-hydroxylase encoded by the cytochrome P450 (CYP) isoform family 2, subfamily R, polypeptide 1 (*CYP2R1*), but this reaction can also be mediated by other CYP isoforms including *CYP27A1*, *CYP3A4* and *CYP2J3*, which results in the formation of 25-hydroxyvitamin D (25(OH)D) [5,6,7]. 25(OH)D has a very long half-life of several weeks and also is one of the major circulating metabolites, which is used to measure vitamin D status in humans [2]. The second step in metabolism is mainly in the kidneys, in which the 1α-hydroxylation (mediated by *CYP27B1*) occurs and is stimulated by the calcium/phosphorus regulatory hormone, parathyroid hormone (PTH) [1]. Conversion by *CYP27B1* generates the most active metabolite, 1,25-dihydroxyvitamin D_3_ (1,25(OH)_2_D_3_) [2,8]. 1,25(OH)_2_D_3_ strongly induces gene expression of *CYP24A1* to produce the enzyme 25-Hydroxyvitamin D_3_-24-hydroxylase that initiates catabolic degradation, resulting in the formation of 1,24,25(OH)_3_vitamin D_3_ and ultimately in the formation of 1α-hydroxy-23-carboxy-24,25,26,27-tetranorvitamin D_3_ [9]. This enzyme also promotes the formation of 24,25(OH)_2_ vitamin D_3_ via negative feedback by decreasing the 25(OH)D substrate available for 1α hydroxylation [9,10]. 1,25(OH)_2_D_3_ has different functions including regulation of intestinal calcium and phosphate absorption, calcium mobilization from bone, and reabsorption of calcium in the kidney. It also has different immune effects in the body [1,11]. 1,25(OH)_2_D_3_ binds to the vitamin D receptor (VDR), which is a member of the superfamily of nuclear receptors for steroid hormones [12,13,14]. The VDR complex can interact with different gene transcription factors leading to both activation and repression of genes that control inflammatory responses [15,16]. VDR can be activated by nanomolar concentrations of a ligand [17]. The nuclear receptors for the steroid hormones estradiol (ERα and ERβ), androgen receptor (AR), progesterone receptor (PR), glucocorticoid receptor (GR) and mineralocorticoid receptor (MR) also share this property, as well as for the vitamin A derivative all-*trans* retinoic acid receptors (RARα, RARβ and RARγ) and for the thyroid hormone triiodothyronine (TRα and TRβ) [18,19]. VDR binding can also be facilitated by the transcription factor activator protein 1 (AP1) [20]. Other transcription factors including Forkheadbox protein A1 (FOXA1) or the hematopoetic transcription factor PU.1 encoded by the *Spi-1* proto-oncogene (SPI-1) can act as pioneer factors for the VDR [18]. VDR agonists can act as an immunosuppressive molecule that can promote the intrinsic tolerogenic capacity of dendritic cells (DCs) in mouse and humans [21,22]. Given the evidence that VDR is expressed in many immune cells, including monocytes/macrophages, B and T cells [10,23,24,25,26] as well as DCs, along with the ability of DCs to produce 1,25(OH)_2_D_3_ [25], this review will focus on the function of VDR in dendritic cells.

## 2. Dendritic Cell Subsets

DCs are replenished from bone marrow (BM) precursors, but may also arise from blood monocytes under inflammatory conditions [27]. They play a critical role in the cellular immune response to self and foreign antigens and have a central role in the orchestration of the regulatory elements of immune homeostasis [28,29]. Dendritic cells specialize in capturing, processing, and presenting antigens to the adaptive immune system. Dendritic cells express lymphocyte co-stimulatory molecules, then migrate to lymphoid organs and secrete cytokines for the regulation of immune responses. Furthermore, DCs are important in the development of immunological memory and tolerance [27,30]. In the context of infection or exposure to non-self antigens, these cells can recognize both pathogen-associated molecular patterns (PAMPs), as well as cellular damage via pattern recognition receptors (PRRs). Activation of these receptors on DCs results in increased expression of antigen presentation machinery including the major histocompatibility complex type II (MHC-II) proteins, as well as co-stimulatory molecules [31,32,33,34]. This signaling allows for efficient antigen presentation to T cells followed by promotion and proliferation of distinct T helper (Th) cell subsets [31,32,33,34].

In mice and humans, DCs can be sub-classified based on morphology, origin, function and anatomical location [28,35,36]. Resident DCs are localized in lymphoid tissue (LT), where antigen uptake occurs from the lymph and bloodstream and they present it to local naïve T cells [36,37]. Non-lymphoid tissue (NLT) DCs, constitute cells that reside in tissues, then migrate to the lymph nodes and present antigens derived from mucosal sites to T cells [36]. Dendritic cell populations in the peripheral blood of humans have also been identified based on the human leukocyte antigen-D related (HLA-DR)^+^ lineage found on their surface marker expression [36,38,39]. Studies on human peripheral blood analyzed the transcriptome of classical and non-classical monocytes (CD14^+^CD16^−^ and CD14^+^CD16^+^, respectively) against DCs defined as HLA-DR^+^ positive and negative for markers of other leukocyte lineages [39]. They found that the DCs clustered into three distinct populations with expression profiles clearly unique from both monocyte populations [40]. These DCs have been further classified as Plasmacytoid DCs (pDCs) and two subsets of myeloid DCs (mDCs). In humans, plasmacytoid DCs circulate in the blood and lymph node (LN) compartments and are characterized by CD123 interleukin-3 receptor (IL-3R), CD303 (BDCA-2), and CD304 (BDCA-4 or Neuropilin-1) expression [28]. The two myeloid DC (mDC) subsets are also referred to as conventional DCs (cDCs) and are identified by their surface markers: CD1c+/BDCA-1^+^ (CD1c^+^ cDC) or CD141^+^/BDCA-3^+^ (CD141^+^ cDC). The total blood DC population consists of about 5%–10% CD141^+^ cDCs, and the rest divided equally among pDCs and CD1c^+^ cDCs [28,36,41]. These subsets can also be found in the spleen and tonsils, however, it has not been reported in humans if there are differences in VDR expression among these subsets [42]. It has been demonstrated that some human DC subsets are also found in the mouse [36,40,43]. Comparison of the gene expression patterns using cross-presentation assays of all known human and mouse DC subsets revealed the following similarities as described in Table 1: human blood pDCs are equivalent to mouse pre-conventional DCs (pre-cDCs), CD141^+^ cDC are comparable to mouse CD8α^+^ DC, and human CD1c^+^ cDC are comparable to mouse CD11b^+^ DC [36,40,43,44,45]. Human analysis of NLT DCs in the skin, lung, and liver identified two cDCs subsets identical to CD1c^+^ and CD141^+^ blood cDCs. Furthermore, this study showed that pDCs were absent in skin, lung and liver in humans under steady-state conditions [46,47]. Transcriptome analysis comparing human and mouse DCs found that human CD1c^+^ and CD141^+^ tissue-resident DCs correspond to mouse NLT DCs, CD11b^+^ and CD103^+^ DCs, respectively [46,47].

**Table 1:** DC development, subsets and lineage-specific markers.

Dendritic cells	Location	Human		Mice	
		Alternative Subset Name	Surface Markers	Alternative Subset Name	Surface Markers
Conventional DCs (cDCs)	Myeloid (Blood)	CD1c/BDCA-1+	CD1c+, CD11c+++, CX3CR1+, CD172a+, CD64+	CD11b^+^/CD103^+^	CD103 integrin marker (aE b7), IRF8
		CD141^+^/BDCA-3+	CD141^+^, CD11c+++, CLE9A+, XCR1+, BDCA-3+	CD8+	CD8α+, NECL2 (CADM1), CLE9A, BATF3, XCR1
	Non-lymphoid tissue (NLT) skin, liver, lung and intestine	CD1c+	CD11c++, CD1c+, CD172a+, CD11b+, CD206+, CD64+, Lower expression of FLT3 and CLEC9A and intermediatelevels of M-CSFR and CX3CR1, compared with CD141^+^	CD11b+/CD103+	CD11c++, CD11b++, CD103 integrin marker (aEb7), CD24++, CD209a+, IRF8
		CD141^+^/CLEC9A+	CD11c+, XCR1+, TLR3, CLEC9+, CD141+, CADM1, CCR7	CD11b+/CD103	CD11c++, CD103 integrin marker (aEb7), CD24+, XCR1+, IRF8
		CD141^+^	CD11c++, CD141+, CX3CR1+, CD1c+, CD172a+, CD11b+, CD206+, CD14+		
	Lymphoid tissue (LT)	CD141^+^	CD141+, CD11c++, CLEC9A+, XCR1+	CD11b+	CD4, Endothelial cell-selective adhesion molecule (ESAM), EB12
		CD1c^+^	CD1c+, CD11c+++	CD8a-	CD11c++, CD11b++, CD4, SIRPa+, DCAL2, Clec 12a, CD209a+, F4/80+
		CD11c^+^	CD123 (IL-3R), CD303 (BDCA-2), CD304 (BDCA-4 or Neuropilin-1)	CD8a+	CD8++, CD11c++, CD11b++, CD103+, CD86+, CD24+, Xcr1+, TLR3, T. gondii sensor, TLR11
Plasmacytoid (pDcs)	Blood and lymph node (LN)	CD11c^+^	CD123 (IL-3R), CD303 (BDCA-2), CD304 (BDCA-4 or Neuropilin-1)	Pre-conventional DCs (pre-cDCs)	PDCA-1
Langerhans cells (LCs)	Epidermal	CD1a	Langerin (CD207+), CD11c+, BDCA1+, CD172a+, CD11b+, CD1a++, E-cadherin+, CD326+, XCR1, CSF1R	LCs	CD11c+, Langerin (CD207+), XCR1+
DCs	Dermal	CD1a+ CD14−	CD1a+ CD14−	CD103+CD207+	IRF8, ID2, BATF3, CLEC9A, XCR1
				CD103+CD207+	
		CD14+	M-CSFR, CX3CR1, CD209 (DC-SIGN)	CD207− CD11b+	
				CD207−, CD11b−, CD103−	

DCs can also reside in the dermis of human skin and represent a large subset of dermal DCs involved in tissue homeostasis [48]. The human skin has three main cutaneous DC populations: epidermal Langerhans cells (LCs), CD1a^+^CD14^−^ dermal DCs and CD14^+^ dermal DCs as shown in Table 1 [48,49]. In humans, LCs highly express the non-classical MHC class I molecule CD1a [50,51]. CD14^+^ dermal DCs express a prominent “mixed” DC/macrophage phenotype [46]. CD14^+^ dermal DCs express low levels of CD80 and CD86 and are poor inducers of naïve T-cell proliferation [52,53], however they can efficiently take up antigen [54] and they can induce CD25+ T regulatory cells (Tregs) through production of interleukin-10 (IL-10) [55]. CD141^+^ mDCs are less immunogenic and may be able to differentiate into the Langerhans cells of the skin in response to transforming growth factor β (TGF-β) [56,57]. In vitro, human CD141^+^ dermal DCs are efficient at cross-presenting soluble antigens as compared to other interstitial DCs and epidermal LCs.

In mice, mature DCs show a high level of expression of MHC II and the co-stimulatory molecules CD80 and CD86 and induce differentiation of naive CD4+ T cells, while immature DCs with low expression of these molecules are more endocytic and efficient at antigen processing [58,59]. mDCs produce high levels of interleukin-12 (IL-12), whereas pDCs have the ability to quickly produce high levels of type I interferons-α (IFN-α) [36,60] in response to viral infections in humans [61]. In response to bacterial and viral stimulation, human pDCs and mDCs produce different patterns of chemokines [27,62]. mDCs preferentially produce very high levels of the chemokine ligand 17 (CCL17) and chemokine ligand 22 (CCL22), whereas pDCs show minimal production of these chemokines [15]. pDCs can produce the pro-inflammatory chemokine ligand 3 (CCL3), whereas chemokine ligand 4 (CCL4) and chemokine ligand 8 (CCL8) can be produced by both subsets [27,62]. pDCs express endosomal toll-like receptors (TLR) 7, 8, and 9 which are able to detect nucleic acids derived from viruses, bacteria, and unmethylated CpG sequences in DNA molecules respectively. In humans, activation of TLR7 or TLR9 triggers a signaling cascade and upregulates the expression of interferon-α (IFN-α), interferon-β (IFN-β) [63] and interferon-λ (IFN-λ) [64]. pDCs contribute to the rapid and large amount of type I IFN production in response to viral infection and are critical in anti-viral immmunity [65].

## 3. Effects of Vitamin D on DC Function

### 3.1. DCs Maturation–Co-Stimulation

The modifications in phenotype as well as the functional plasticity of DCs varies through multiple signal codes that are generated by different stimuli in humans [66]. There are two major phases in the life of DCs, an immature stage, which is highly effective in terms of antigen uptake and processing, and the mature stage where the antigen uptake capacity is lost and the cell migrates toward regional lymph nodes, shifting the function to become a potent antigen-presenting cell (APC) [67].

1,25(OH)_2_D_3_ and VDR can regulate human DC maturation [68]. Exposure of differentiating human and mouse monocytes to 1,25(OH)_2_D_3_ increased the expression of molecules involved in antigen capture and inhibited DC differentiation and maturation, which impaired stimulatory capacity for the antigen specific CD8 T cells [29,69,70,71,72,73]. Furthermore, they stimulate an increase in the number of Tregs, directly affect CD4+ T cells, up-regulate IL-10, as well as reduce tumor necrosis factor-α (TNF-α) and interferon γ (IFN-γ) levels [29,69,70,71,72,73,74]. These molecular changes may play a role in the inhibition and interaction between DCs and T cells in mice and humans [70].

**Figure 1:**
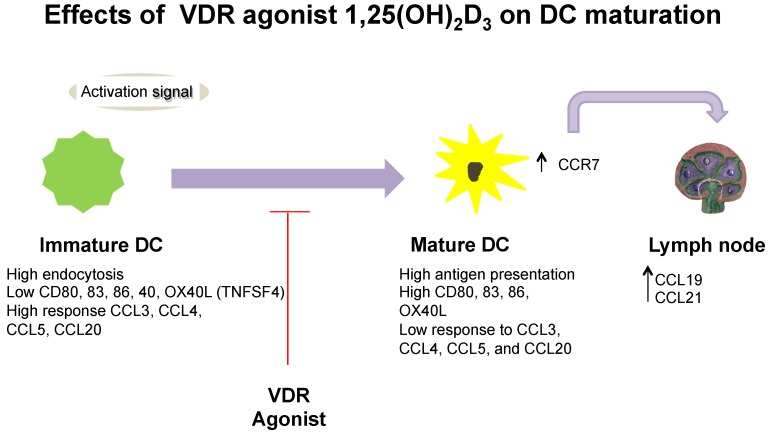
Overview of vitamin D on dendritic cell (DC) function.

*In vivo* and *in vitro* experiments have shown that 1,25(OH)_2_D_3_ can induce mouse and human mDCs that have a tolerogenic phenotype, characterized by decreased CD40, CD80, and CD86, low interleukin-12 (IL-12), and enhanced IL-10 secretion, as shown in Figure 1 [69,75]. Specifically, immature monocyte-derived DCs were generated from buffy coat monocytes, activated by lipopolysaccharide (LPS) and stimulated with 1,25(OH)_2_D_3_, which resulted in the inhibition of pro-inflammatory cytokines such as the heterodimeric molecule interleukin-12p70 (IL-12p70) in both humans and mice [69,72]. The intrinsic production or exogenous stimulation by 1,25(OH)_2_D_3_ can arrest the differentiation and inhibit maturation of mDCs, resulting in the decreased expression of maturation markers CD40, CD80, CD86 and retention of antigen uptake, as shown in Figure 1 [29,72,76,77]. The expression of maturation markers CD40, CD80 and CD86 were inhibited, along with decreased IL-12 and upregulation of IL-10 production after mDCs were stimulated with 1,25(OH)_2_D_3_, and furthermore, 1,25(OH)_2_D_3_ stimulation led to decreased activation of CD4+ T cells in humans [21] and an increase in iTreg cells in mice [78]. Using genetic approaches to study VDR function, VDR-deficient mice compared with wild-type mice were found to demonstrate subcutaneous lymph node hypertrophy with an increase in mature DCs [76]. In addition to these effects, 1,25(OH)_2_D_3_ has marked effects by suppressing chemokines CCL17 and CCL22 in human mDCs, as shown in Figure 1 [79,80]. There are two pathways that can explain the anti-inflammatory effects of 1,25(OH)_2_D_3_ in mDCs [16,81]. In early inflammation, 1,25(OH)_2_D_3_ has a primary and direct effect on the up-regulation of the chemokine ligand (CXCL) gene expression via direct binding of VDR to the CXCL cluster locus in humans [16]. For the latter phase of inflammation, the secondary effect may demonstrate an overreactive inflammatory response that is controlled by 1,25(OH)_2_D_3_ via the repression of the transcription factor nuclear factor kappa-light-chain-enhancer of activated B cells (NF-κB) [16,81]. In mice, DCs can be mobilized from the skin to the draining LN in response to a subcutaneous latex microsphere injection, but when 1,25(OH)_2_D_3_ is added to the microsphere inoculum, the DCs bypassed the draining LN and enter non-draining secondary lymphoid organs, including Peyer’s patches [82]. Furthermore, measurement by surface phenotype of the microsphere in human and murine myeloid/conventional DCs exposed to 1,25(OH)_2_D_3_ showed reduced chemotaxis toward a chemokine receptor 7 (CCR7) and chemokine ligand 21 (CCL21) [80,83], which are required for DC emigration from inflamed tissues to draining lymph nodes [79,80,83]. A similar effect has been observed in LCs where stimulation with 1,25(OH)_2_D_3_ decreased the chemotaxis of LCs towards the chemokine ligand 21 (CCL21), likely due to the inhibition of CCR7 expression, as shown in Figure 1 [84].

Immunoglobulin-like transcript 3 (ILT3) expression by DCs is required to induce CD4^+^Foxp3^+^ regulatory T cells [77,80,85]. One study found that 1,25(OH)_2_D_3_ was able to induce up-regulation of ILT3 expression on immature and mature human DCs [85]. Furthermore, NF-κB activity has been shown to regulate the production of IL-12, type I IFNs, CCL7, chemokine receptor 22 (CCL22), and the expression of MHC class II molecules, CD40, CD80, CD86, and ILT3 [15]. NF-κB is a regulator of the immune system, inflammatory genes and is also a target for many anti-inflammatory and immunosuppressive agents [69], including glucocorticoids and anti-inflammatory medications that bind to the same family of nuclear receptor as the VDR [86,87]. Human mDCs treated with 1,25(OH)_2_D_3_ showed decreased nuclear translocation of the p65 subunit of NF-κB, which may explain some of the anti-inflammatory effects of 1,25(OH)_2_D_3_ [80].

Type I IFN mediates and induces the differentiation of monocytes to DCs (type 1 IFN DCs). Freshly isolated monocytes treated with 1,25(OH)_2_D_3_, inhibited the generation of type 1 IFN DCs [88]. Monocytes that were freshly isolated and cultured with GM-CSF and IFN-β along with 1,25(OH)_2_D_3_ compared with control IFN-DCs, showed that IFN-DCs cultured in the presence of 1,25(OH)_2_D_3_, failed to up-regulate the differentiation marker CD1a or the maturation marker CD83 [88]. IFN-DCs also had significantly impaired functional activities. For example, IFN-DCs exhibited a potent allostimulatory capacity, while cells cultured with 1,25(OH)_2_D_3_ had limited capability to stimulate T cell proliferation [88]. Additionally, when 1,25(OH)_2_D_3_ was added to human IFN-generated DCs, these cells could not produce interleukin-1α (IL-1α) and demonstrated impaired chemotaxis in response to both CCL4 and CCL19 [88,89].

As stated above, pDCs are major producers of type 1 interferon especially following viral infection [60]. 1,25(OH)_2_D_3_ treatment of pDCs resulted in no effect on T helper 1 (Th1) development or Treg activity [80]. All-*trans*-retinoic acid (RA) plays a critical role in maintaining intestinal immune homeostasis [90]. Experiments demonstrated that human blood CD1c^+^ mDCs, but not CD141^+^ mDCs or plasmacytoid DCs responded to 1,25(OH)_2_D_3_ by promoting the production of RA by highly expressing retinaldehyde dehydrogenase (RALDH2) mRNA and aldehyde dehydrogenase (ALDH) activity [91]. RALDH2 is an enzyme that converts retinol to retinoic acid and promotes CD4^+^ T cells to acquire the ability to produce T helper 2 (Th2) cytokines in an RA-dependent and an IL-4-independent manner [92]. Murine experiments also demonstrated that CD103^+^ DCs found in the lamina propria and mesenteric lymph nodes (MLNs) can produce RA and promote the conversion of naïve T cells to Foxp3^+^ T regulatory cells in the intestine, contributing to the maintenance of intestinal immune homeostasis [93].

### 3.2. DC Cytokine Production

**Figure 2:**
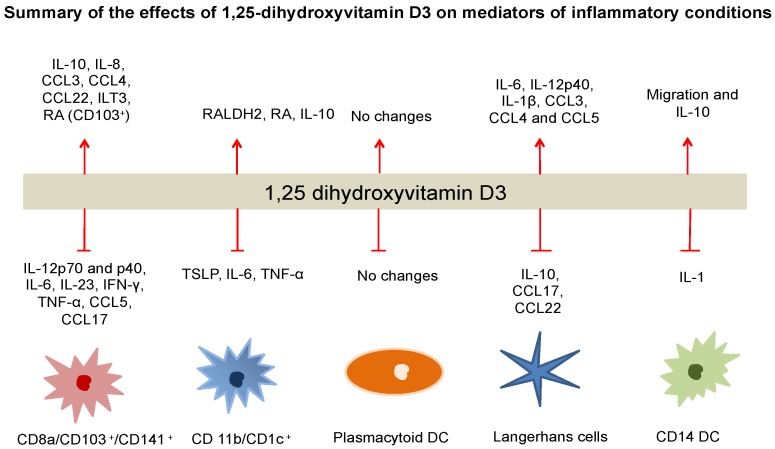
The influence of 1,25(OH)_2_D_3_ on the expression of interleukins, cytokines and regulatory molecules in different DC subsets.

Studies have shown that DCs stimulated with 1,25(OH)_2_D_3_ showed increased IL-10 production and an inhibition of the release of pro-inflammatory cytokines including TNF-α, IFN-γ and IL-12 [74,77,94,95]. It has been demonstrated that IL-12 production in DC cultures is inhibited by intrinsic 25(OH)D conversion in humans [70]. IL-12 is important to the induction of Th1 responses via inhibition of TNF-α production [70]. The expression of CD14, which is a monocyte/macrophage marker, was increased after 1,25(OH)_2_D_3_ exposure, indicating that the developing DC differentiates towards a more macrophage-like and less antigen-presenting pathway [70]. By a functional assay, 1,25(OH)_2_D_3_ reduced the ability of DCs to induce proliferation of human tetanus toxoid (TT)-specific T lymphocytes [70]. Human blood derived mDCs exposed to 1,25(OH)_2_D_3_ showed inhibition of CD40-triggered production of both interleukin-12p70 (IL-12p70) and interleukin-12p40 (IL12-p40), but not IL-10 [96]. The resultant inhibition of the IL-12p40 chain, which heterodimerizes to form both IL-12 and IL-23, resulted in a marked reduction in the development of Th1 and T helper 17 (Th17) cells [80]. Mouse LCs exposed to 1,25(OH)_2_D_3_ demonstrated up-regulation of the production of IL-1β, CCL3, CCL4 and CCL5 [84]. 1,25(OH)_2_D_3_ stimulation also affects mDCs, by inhibiting the development of Th1 and favoring Th 2 induction, resulting in transcriptional repression of IL-2 and IFN-γ [74,75,97]. Human myeloid DCs stimulated with 1,25(OH)_2_D_3_ showed up-regulation in the expression of CCL3 and CCL4, but down-regulation of CCL5 [98,99]. LCs exposed to 1,25(OH)_2_D_3_ had suppressed production of Th 2 type chemokines, CCL17 and CCL22 upon activation through CD40 ligation, which are largely secreted during LC maturation [80]. Chemokines such as CCL22 were up-regulated, whereas the CCR4 ligand, CCL17 was down-regulated by 1,25(OH)_2_D_3_ in mDCs as shown in Figure 2 [80].

There have been several polymorphisms of the human VDR gene identified, specifically resulting in VDR proteins with different structures, either a long f-VDR or a shorter F-VDR [100]. Shorter VDR protein of 424 amino acids (aa) or the long isoform with 427 aa have been shown to influence IL-12 expression in DCs [100]. A study evaluating the IL-12 promoter activity in human mDCs showed that the presence of the shorter F-VDR led to an increase in the expression of NF-κB and nuclear factor of activated T-cells (NFAT)-driven transcription, as well as higher IL-12p40 promoter activity [100]. It was also found that the levels of IL-12p35, the other component of IL-12p70, were higher in antigen-presenting cells from F-VDR genotype [100].

The 1,25(OH)_2_D_3_-VDR complex may have effects in different group of cells, including DC interactions with transcription factors such as NF-κB, NFAT, or the glucocorticoid receptor (GCR) leading to anti-inflammatory effects [101,102]. Ubiquitination-mediated proteolysis of nuclear factor of kappa light polypeptide gene enhancer in B-cells inhibitor α (IκBα) by the 26S proteasome is a critical step in NF-κB activation [103]. In a human monocyte-like THP-1 cell line and in DCs upon direct binding of 1,25(OH)_2_D_3_ to VDR, NF-κB activation is inhibited by interactions with a specific inhibitor IκB, which allow NF-κB to remain in the cytosol [15,16]. When there is an inflammatory event that stimulates the cells, IκB gets phosphorylated, ubiquitinated, and subsequently degraded by the IκB kinase [104]. Free NF-κB translocates to the nucleus, where it initiates the transcription of pro-inflammatory cytokines and promotes apoptotic events, as well as activates enzymes involved in pro-inflammatory mediator generation such as cyclooxygenase-2 (COX-2) [105]. This cascade of inflammatory events may be affected by the repressive effects of the 1,25(OH)_2_D_3_-VDR complex on NF-κB. Six ChIP-seq data sets found 21,776 non-overlapping VDR binding sites, whereas only 54 sites were common in all six data sets. This suggests that VDR binding is cell and stimulus-specific. Only 17.5% of the non-overlapping binding sites contain a DR3-type VDRE, whereas the percentage of DR3-type response elements is enriched in highly ligand-responsive loci. These data suggest that the VDR interacts with other transcription factors and that these interactions may be only in part ligand dependent [101,105].

### 3.3. DCs Migration/Antigen Presentation: in Vivo Studies

A murine model of a vaccination with the Toll-like receptor 4 (TLR4) adjuvant Monophosphoryl Lipid A (MPLA) in wild-type (WT) and 1α-hydroxylase (1αOHase)-deficient mice showed that in the presence of 1,25(OH)_2_D_3_, mDCs were incapable of migrating beyond the draining LNs following vaccination [106]. These data suggest that a local production of 1,25(OH)_2_D_3_ is required for the migration of DCs beyond the draining LNs [106]. The presence of 1,25(OH)_2_D_3_ during *in vitro* DC maturation of immature DCs of human monocytes cultured with granulocyte macrophage colony-stimulating factor (GM-CSF) and IL-4 resulted in promotion of spontaneous DC apoptosis [77]. DCs generated from human monocytes showed decreased DC survival along with significantly lower levels of HLA-DR and CD86 [107], affecting the persistence of antigen presentation (measured by flow cytometry) an important prerequisite for proper T cell (re)activation [107]. Moreover, another study reported that DCs had a dose dependent response to 1,25(OH)_2_D_3_, where CD80 and HLA-DR were down-regulated after stimulation with a high concentration of the Vitamin D analogue TX527 (19-nor-14,20-bis-epi-23-yne-1α,25(OH)_2_D_3_) at the highest doses of 10^−7^ M and 10^−8^ M. However, this response was lost when using 1,25(OH)_2_D_3_ at a lower concentration of 10−10 M [68]. 1,25(OH)_2_D_3_ and 25(OH)D-treated LCs and dermal DCs express elevated IL-10 levels and promote the development of IL-10-producing Treg cells in humans [108]. Interestingly, 25(OH)D-treated DCs had persistent production of IL-12 that led to the development of IFN-γ-producing T cells, however vitamin D_3_ had no direct effect on IFN-γ production by T cells and 1,25(OH)_2_D_3_ inhibited IL-12 production [109].

Intradermal 1,25(OH)_2_D_3_ injection influences the different human skin DC subsets, CD1a(+), langerin(+) Langerhans cells, CD14(+) dermal DCs and CD1a(+) and selectively enhanced the migration of CD14(+) DCs, a subset known for the induction of tolerance [82,110]. Furthermore, intradermal 1,25(OH)_2_D_3_ repressed the LPS-induced T cell stimulatory capacity of migrating DCs in mice [110]. These migrating DCs induced T cells with suppressive activity and eliminated IFN-γ productivity and promoted the development of Foxp3(+) Tregs [111]. In addition, the postmigrational DCs, macrophage-like CD14(+)CD1a(−) DC subset showed poor T cell-stimulatory abilities [112]. Mature and immature DCs are not known for their capacity to kill ingested bacteria [113], however, the mixed DC-macrophage phenotype observed after stimulation of CD14(+)/CD1a(−) DCs with 1,25(OH)_2_D_3_, support the finding that 1,25(OH)_2_D_3_ can enhance the production of the human cathelicidin LL-37 [65,114,115].

## 4. Implications for Human Disease

### 4.1. Allergic Disease

Allergic bronchopulmonary aspergillosis (ABPA) is caused by a Th2 immune response to antigens derived from *Aspergillus fumigatus* *(A. fumigatus)*. Patients with ABPA have an increased IL-13 response in blood CD4+ T-cells when stimulated with autologous CD11c^+^ DCs and pulsed with *Aspergillus* antigens [116,117]. Addition of vitamin D3 can suppress this *A. fumigatus*-specific Th2 response in peripheral CD4^+^ T cells in patients with cystic fibrosis (CF) and ABPA [116]. As a result of this specific Th2 suppression, there was an increase in TGF-β^+^ regulatory T cells, and suppression of OX40 ligand (OX40L), a costimulatory molecule on dendritic cells that is regulated by thymic stromal lymphopoietin (TSLP), an epithelial cell cytokine that can drive Th2 differentiation [116,118].

As mentioned above, the 1,25(OH)_2_D_3_-VDR complex can decrease the maturation of DCs and decrease the DCs capacity to activate alloreactive T cells [69,79]. DCs have an important role in initiating and maintaining allergic Th2 immune cell responses to inhaled allergens [50]. CD11c^+^ mDCs also express receptors for TSLP [117,119], and is required for the development of inflammatory allergic responses [120]. TSLP-activated DCs express OX40L through the activation of NF-κB components [66], which is responsible for triggering Th2 inflammation in the lung [118,121]. Blockade of OX40L inhibits antigen-specific Th2 inflammation [122]. Another study reported that in the lungs of vitamin D-deficient mice, their lung CD11c**^+^** DCs have increased expression of OX40L and stimulation with vitamin D_3_ inhibits the promoter activity of OX40L [123]. This study demonstrates that vitamin D_3_ leads to VDR binding to the OX40L promoter and represses OX40L promoter activity [123]. Specifically, this study found that VDR and the p50 and p65 subunits of NF-κB bind to the promoter region of OX40L, which down-regulate the expression of OX40L. In addition, they found that treatment with vitamin D_3_ inhibited OX40L promoter activity that was induced by TNF-α [123].

### 4.2. Autoimmunity

Studies have suggested that Vitamin D status plays an important role in the initiation, progression and/or severity of different autoimmune diseases such as rheumatoid arthritis, multiple sclerosis, asthma, systemic lupus erythematous and inflammatory bowel disease (IBD) [2,124,125,126,127,128,129,130]. IBD such as Crohn’s disease and ulcerative colitis, are chronic, idiopathic inflammatory disorders of the gastrointestinal tract [131]. In the context of IBD, DCs play an important role in directing immunity and regulating intestinal mucosal inflammation by modulation of many cell types including Tregs, Th17 cells as well as natural killer (NK) cells, monocyte and macrophages [132]. Activated and mature DCs in Crohn’s disease induce the production of inflammatory cytokines such as IL-12, IL-18, TNF-like 1A and IFN-γ, which stimulates macrophages to release IL-1α, TNF-α, and IL-6 [133,134].

Additionally, murine studies reported the development of experimental colitis in IL-10 knock-out (KO) mice that were exposed to a Vitamin D-deficient diet. Vitamin D-deficient IL-10 KO mice start dying at 7 weeks of age and by 9 weeks of age, 58% (15/26) of the vitamin D-deficient IL-10 KO mice were dead [135]. After 9 wks of age, the remaining vitamin D-deficient IL-10 KO mice had persistent weight loss [135]. In contrast, the vitamin D-sufficient IL-10 KO (*n* = 10) and the vitamin D-deficient WT mice (*n* = 20) appeared healthy, even up to 13 wks of age [135].

VDR activation by the intrinsic production of 1,25(OH)_2_D_3_ in type 1 IFN DCs, macrophages and intestinal epithelial cells, can promote transcription of the Nucleotide-binding oligomerization domain protein 2/caspase recruitment domain-containing protein 15 (NOD2/CARD15), a cytosolic protein involved in intracellular recognition of microbes by sensing peptidoglycan fragments (e.g., muramyl dipeptide) [136,137,138]. VDR activation by the intrinsic production of 1,25(OH)_2_D_3_ in monocyte-derived cells and epithelial cells promoted the transcription of NOD2 and expression of genes encoding for antimicrobial peptide defensin β2 (DEFB2)/Human β-defensin-2 (HBD2) and the antimicrobial cathelicidins in the presence of muramyl dipeptide [139]. This signaling pathway was defective in cells expressing the major variant of NOD2 present in a subset of patients with Crohn’s disease [140]. NOD2 may affect the intestinal microbiome and can also potentiate autophagy, which is the process by which damaged organelles, proteins and intracellular microorganisms are removed through engulfment into an autophagosome and are then degraded by lysosomes [141]. Intestinal microbiota is a main driver for the development of the mucosal immune system. A dysregulated immune response to infection may cause perturbations of this interaction with the intestinal microbiota that may lead to disorders such as IBD [142,143,144,145]. Immune responses elicited by intestinal DCs induce anti-inflammatory and tolerogenic responses to harmless antigens such as those derived from the resident microflora in mice [146]. 1,25(OH)_2_D_3_ can activate signaling programs in DCs that yield in priming of regulatory and anti-inflammatory T cell responses [146]. Murine vitamin D deficiency results in the overproduction of Th1 and Th17 immune responses [147] and a reduction in the amount of tolerogenic DCs and regulatory T-cells [148]. Vitamin D and the VDR inhibit Th1, Th17, and inflammatory cytokine production in the gastrointestinal tract that serve to reduce inflammation, shift the microbiome, and maintain tolerance within the intestine [146]. Additional functions of 1,25(OH)_2_D_3_ and VDR in IBD include functioning as a regulator of T cell function, which has been reported specifically as having the ability to turn off chronically activated T cells [149]. Other additional roles of 1,25(OH)_2_D_3_ and VDR include providing protection in mucosal barrier homeostasis by contributing to the maintenance of the integrity of the tight junction proteins zonula occludens-1 and claudin-1 in mice [146]. In addition, 1,25(OH)_2_D_3_ also contributes to the healing of the colonic mucosa [150,151] and maintenance of the gut microbiome [146]. A recent study reported dysbiosis in different mice fed a Vitamin D-deficient diet, VDR knock out (VDR KO) mice, and *Cyp27b1* knockout (Cyp KO) mice [146]. Cyp KO and VDR KO mice had more bacteria from the *Bacteroidetes* and *Proteobacteria* phyla and fewer bacteria from the *Firmicutes* and *Deferribacteres* phyla in the feces compared with wild-type mice. There was an increase in the *Helicobacteraceae* family in Cyp KO compared with wild-type mice [146]. This study also showed that depletion of the gut bacterial flora using antibiotics protected mice from colitis [146]. Providing 1,25(OH)_2_D_3_ treatment (125 μg/100 g diet) to Cyp KO mice decreased colitis severity and reduced the numbers of *Helicobacteraceae* in the feces compared to the feces of untreated Cyp KO mice [146]. The mechanisms by which the dysbiosis occurs in VDR KO and Cyp KO mice included lower expression of E-cadherin on gut epithelial cells and immune cells as well as fewer tolerogenic dendritic cells, resulting in more gut inflammation in VDR and Cyp KO mice compared with wild-type mice [146]. Several studies suggest that Vitamin D has a potential role in the therapy of IBD [152,153,154,155]. Randomized controlled trials have reported that patients with IBD may remain in remission longer when treated with oral treatment with 25(OH)D 1200 IU daily [152]. Suboptimal Vitamin D status is common in IBD, and studies suggest that this factor is associated with increased disease severity [156]. The 1,25(OH)_2_D_3_ anti-inflammatory role has been reported and one of the recommendations in the clinical management of Crohn’s disease is to prevent Vitamin D deficiency [153].

## 5. Summary/Perspective

The active form of vitamin D, 1,25(OH)_2_D_3_ has in addition to its central role in calcium and bone metabolism, has important immunoregulatory and anti-inflammatory effects. This secosteroid hormone affects the growth, differentiation and molecular expression of many cell types. The biological effects of 1,25(OH)_2_D_3_ are mediated by the extrinsic or intrinsic cell activation or 1,25 hydroxylation of vitamin D_3_. The VDR is a member of the superfamily of nuclear hormone receptors. 1,25(OH)_2_D_3_-VDR complex formation leads to interactions with various transcription factors within the immunomodulatory response and is reported to have anti-inflammatory and allogenic effects. VDR is present in most cell types of the immune system, in particular in antigen presenting cells (APCs) such as macrophages and DCs, as well as in both CD4^+^ and CD8^+^ T cells. DCs have an important role in capturing and processing antigens; they express lymphocyte co-stimulatory molecules, migrate to lymphoid organs and secrete cytokines to initiate immune responses. Specifically, the 1,25(OH)_2_D_3_-VDR complex affects the maturation and migration of many subsets of DCs, conferring a special immunoregulatory role along with tolerogenic properties affecting cytokine and chemokine production. These vitamin D_3_ immunoregulatory activities have been an intense area of investigation in allergic and autoimmune diseases and it remains to be determined if these activities are directly related to serum 25-OH vitamin D levels which are currently being used to assess vitamin D sufficiency. Additional basic studies as well as well-designed clinical studies will clarify the role of vitamin D_3_ in DC function in humans.

DCs are a heterogeneous population of immune cells and DC precursors develop in the bone marrow. While plasmacytoid DCs complete development in the bone marrow, most DCs complete development in lymphoid and peripheral tissues. DCs can be sub-classified based on morphology, origin, function and anatomical location. Several phenotypic and functional DCs subsets have been identified based on the HLA-DR^+^ lineage found on their surface marker expression including mDCs which are also known as cDCs, pDCs, inflammatory or monocyte-derived DCs (moDCs), LCs and two dermal DCs subsets. Three subsets of DCs have been identified in human blood and tissues that are either CD1c/BDCA-1^+^ or Thrombomodulin/CD141/BDCA-3^+^ mDCS, CD123 (IL-3R), CD303 (BDCA-2) mDCS, and CD304 (BDCA-4 or Neuropilin-1) pDCs. There is functional homology between human and mouse DCs. Multiple cDC subsets have been identified in mice including CD4^−^CD8^+^ cDCs, CD4^+^CD8^−^ cDCs, CD4^−^CD8^−^ cDCs, Integrin alpha E/CD103^+^ cDCs, and Integrin alpha M/CD11b^+^ cDCs. Inflammatory or moDCs develop from monocytes at sites of inflammation and are identified by their expression of Ly-6C in mouse. Langerhans cells can be identified in both human and mouse by the presence of Langerin/CD207-containing Birbeck granules. Two subsets of human and mouse dermal-resident DCs have also been characterized that are defined by the presence or absence of CD14 in human or Langerin/CD207 in mice. Unlike other DCs, pDCs are inefficient antigen-presenting cells and have low MHC class II expression.

Macrophages, DCs and T cells can synthesize 1,25(OH)_2_D_3_ and contribute to the regulation of immune responses. VDR activation by 1,25(OH)_2_D_3_ stimulation or intrinsic hydroxylation of 25(OH)D arrests DC maturation induced by different stimuli, maintaining them in an immature state, in terms of phenotype and functional plasticity. VDR agonists have the capacity to inhibit expression of surface co-stimulatory molecules (e.g., CD40, CD80, CD83 and CD86) and MHC class I and II molecules in several DC subsets including mDCs, cDCs and LCs. 1,25(OH)_2_D_3_-VDR complex inhibits the promoter activity of OX40L or tumor necrosis factor (ligand) superfamily, member 4 (TNFSF4) in CD11c^+^ DCs.

The 1,25(OH)_2_D_3_-VDR complex can inhibit the expression of IL-12, IL-23, IL-6, TNFα and INF-γ, CCL5 and CCL17 in both mDCs and cDCs. In contrast, IL-10 and IL-8 expression can be enhanced by 1,25(OH)_2_D_3_. A shift from a Th1 profile towards a Th2 type and a decrease in Th17 responses is to be anticipated from these changes. In contrast, minimal immunomodulatory effects seem to be exerted by 1,25(OH)_2_D_3_ on circulating plasmacytoid DCs. In mDCs, the expression of surface inhibitory molecules such as ILT3 and of inhibitory cytokines such as IL-10, were markedly upregulated. DCs expressing high levels of inhibitory molecules, such as ILT3, favor induction and/or enhancement of regulatory/suppressor T cells. CD1c^+^ and CD103^+^ mDCs but not CD141^+^ mDCs or plasmacytoid DCs responded to 1,25(OH)_2_D_3_ by promoting the production of retinoic acid (RA) by highly expressing RALDH2. RA production can promote the conversion of naïve T cells to Foxp3^+^ T regulatory cells. Divergent responses have been observed in LCs, such as the expression of IL-10 was down-regulated, but the expression of IL-6 and IL-12p40 was up-regulated, leading to decreased production of Th2 type chemokines, CCL17 and CCL22. In CD14^+^ DCs, the expression of IL10 was upregulated, but IL-1 expression was down-regulated, enhancing the migration of these DCs.
